# Targeting Immune-Related Molecules in Cancer Therapy: A Comprehensive* In Vitro* Analysis on Patient-Derived Tumor Models

**DOI:** 10.1155/2019/4938285

**Published:** 2019-02-12

**Authors:** Claudia Maletzki, Philine Scheinpflug, Anika Witt, Ernst Klar, Michael Linnebacher

**Affiliations:** ^1^Department of Medicine, Clinic III-Hematology/Oncology/Palliative Care, Germany; ^2^Molecular Oncology and Immunotherapy, Department of General Surgery, Germany; ^3^Department of General Surgery, Rostock University Medical Centre, 18057 Rostock, Germany

## Abstract

This study investigated the impact of immune-related pathway inhibition, among them indolamine 2,3-dioxygenase (IDO), alone and together with immune cells on growth and viability of colorectal cancer (CRC) cells. A panel of patient-derived CRC cell lines with different molecular characteristics (CpG island methylator phenotype, chromosomal, and microsatellite instability) was included. Initial phenotyping of CRC cell lines (n=17) revealed high abundance of immunosuppressive checkpoint-molecules in general, but an individual profile for IDO. Presence of immune-related molecules was independent of the molecular subtype. Selective treatment of CRC cell lines showing high or low IDO expression (n=2 cell lines each) was performed with single agents and combinations of Indoximod, Curcumin, and Gemcitabine with and without the addition of peripheral blood lymphocytes (PBL) in an allogeneic setting. All substances affected CRC cell growth in a cell line specific manner. The combination of Curcumin and Gemcitabine proved to be most effective in tumor cell elimination. Functional read-out analyses identified cellular senescence, after both single and combined treatment. Curcumin alone exerted strong cytotoxic effects by inducing early and late apoptosis. Necrosis was not detectable at all. Addition of lymphocytes generally boosted antitumoral effects of all IDO-inhibitors, with up to 80 % cytotoxicity for the Curcumin treatment. Here, no obvious differences became apparent between individual cell lines. Combined application of Curcumin and low-dose chemotherapy is a promising strategy to kill tumor target cells and to stimulate antitumoral immune responses.

## 1. Introduction

Immune-checkpoint inhibitors constitute one of the most promising novel therapeutic approaches for cancer [1]. These molecules reconstitute the hosts' antitumoral immune response by interrupting tumor-induced tolerance and are now at the forefront of immunotherapy development. Unlike great advances in some tumor types including melanoma and non-small cell lung cancer, immunotherapy of colorectal cancer (CRC) remains challenging due to the broad clinicopathological and molecular heterogeneity [2]. Three molecular pathways have been implicated in colorectal tumorigenesis: chromosomal instability (CIN, ~60 %), CpG island methylator phenotype (CIMP, ~30 %), and microsatellite instability (MSI, ~15 %). This latter subgroup is more likely to respond to immunotherapy [3]. An ultrahigh mutational load due to accumulating insertions/deletions in short repetitive sequences (=microsatellites) constitutes the underlying molecular mechanism and* Vice versa*; their high immunogenicity forces MSI^+^ tumor cells to escape the otherwise effective immune attack by creating an immunosuppressive microenvironment. Additionally to downregulate major histocompatibility complex (MHC) class I on the tumor cells' surface and inactivation of the antigen-processing machinery, upregulation of immune-checkpoint-molecules (such as programmed cell death 1 (PD-1)/programmed cell death 1 ligand 1(PD-L1)) represents another escape mechanism [4].

Currently, many immune-checkpoint-molecules, including indoleamine 2,3-dioxygenase (IDO), are exploited as tumor-targeting strategies. IDO is an enzyme of the tryptophan-catabolism and has been implicated in tumor progression. By decreasing tryptophan and increasing kynurenine levels in the tumor microenvironment, IDO effectively inhibits T-cell proliferation and response [5–8]. Indoximod [1-methyl-D-tryptophan] is a small molecule developed to block this IDO-mediated tolerance in order to restore antitumoral immune function [9]. Several clinical trials investigate the potential of Indoximod and other IDO-inhibitors in combination with cytostatic therapy (e.g.,* clinical trials.gov,* identifier: NCT02077881, NCT02052648, and NCT02835729). Recently published phase I studies not only confirm safety (up to 2,000 mg orally twice/day) but also report stable disease for >4 months in some heavily pretreated patients with metastatic malignancies [10–12].

Polyphenols like Curcumin, produced in rhizomes of* Curcuma longa*, are likewise under investigation as additives to immune-based therapies [13, 14]. Antiproliferative effects on CRC cells were reported [15, 16]. Interestingly, Curcumin interacts with IDO to induce natural killer (NK) and T-cell proliferation [17, 18], making this compound another promising candidate for combined approaches.

Some antineoplastic drugs have also been described to interfere with IDO, especially Gemcitabine. As a nucleoside analog and standard drug for pancreatic cancer patients, Gemcitabine not only inhibits cell proliferation but also enhances the activity of NK cells via induction of immunogenic cell death [19, 20].

In this report, we analyzed mechanisms of direct and indirect IDO-inhibition in a panel of molecularly well-characterized patient-derived CRC cell lines. Besides direct impacts on CRC cells, we observed effects of IDO-inhibiting therapies in coculture experiments with immune cells.

## 2. Material and Methods

### 2.1. Tumor Cell Lines and Treatment

Patient-derived HROC cell lines (nomenclature: HRO, Hansestadt Rostock; C, Colon; T, transfer; M, mouse) were established and characterized in our lab [18, Table 1 and* unpublished*]. Authenticity for these lines was verified by means of short tandem repeat fingerprinting (comparison of cell lines at different passages, matched normal tissue, as well as corresponding B cells) from genomic DNA according to [21]. For analyses, cells in passages <40 were maintained in complete medium: DMEM/F12 supplemented with 10 % fetal calf serum, glutamine (2 mmol/l) and antibiotics (PAN-Biotech GmbH, Aidenbach, Germany). Cells were seeded at the appropriate density for each cell line and were incubated 24 h prior to treatment. For all* in vitro* experiments, the following substances and their combinations were used in these concentrations: 11.5 *μ*M Indoximod, 1 *μ*M Gemcitabine, and 20 *μ*M Curcumin. Antitumoral effects were examined after 72 h of incubation.

### 2.2. Phenotyping of Immune-Checkpoint-Molecules via Flow Cytometry

Tumor cells were stained with fluorescently-labeled monoclonal anti-human antibodies (extracellular: PD-L1, PD-L2, B7-H3, B7-H4, CD270, 4-1BBL, OX40L, CD27L, CD40L, CD80, CD86, MHC I, MHC II 1 *μ*g antibody/0.8x10^6^ cells, incubation: 30 min, 4°C; intracellular: IDO1, CD152 (BioLegend®, San Diego, USA) 5*μ*g antibody/0.8 x 10^6^ cells, incubation: 60 min, 4°C after permeabilization: 20 min, 4°C, washed with 1x PBS and analyzed by using the BD FACS Verse™ and BD FACS Suite software application (BD, Heidelberg, Germany).

### 2.3. Functional Analysis of Substance-Mediated Growth Inhibition via Crystal Violet Staining

All experiments were performed in 96-well plates in triplicate and replicated at least three times. Treated cells were stained with crystal violet (0.2 %, 10 min, RT). After adding sodium dodecyl sulfate (1 %) the absorbance was measured at 570 nm (Tecan Trading AG, Männedorf, Switzerland). For sequential treatment, cells were incubated for 72 hours with Indoximod. After removal of media, the cells were incubated for another 72 hours with the test substances and combinations prior to crystal violet staining as described before. Finally, drug effects from triplicate wells were determined in comparison to untreated controls (=set to be 100 %), measured at 570 nm (reference wavelength: 620 nm).

### 2.4. RNA Isolation, cDNA Synthesis, and Quantitative Real-Time PCR

RNA from treated cells was isolated with “Gene Matrix Universal RNA Purification Kit” from EURX® (Gdansk, Poland) according to the manufacturers' instructions. RNA was reverse-transcribed into cDNA from 1 *μ*g RNA using 1 *μ*l dNTP mix, 1 *μ*l oligo (dT)15 Primer, 1 *μ*l reverse transcriptase, and 4 *μ*l 5x RT buffer complete (all purchased from Bioron GmbH, Ludwigshafen, Germany). A volume of 20 *μ*l was filled with RNAse free water. cDNA synthesis conditions were as follows: 25°C for 10 min, 37°C for 120 min, and 85°C for 5 min. Target cDNA levels of human cell lines were analyzed by quantitative real-time PCR using TaqMan Universal PCR Master Mix and predesigned TaqMan gene expression assays for serine/threonine-specific protein kinase Ataxia Telangiectasia Mutated (*ATM)*,* CCNE1 (encoding the cyclin E1 protein)*, cyclin-dependent kinase inhibitor 2A (*CDKN2A)*, Murine double minute 2 (*MDM2)*, and glyceraldehyde-3-phosphate dehydrogenase (*GAPDH;* housekeeping gene as control) in the light cycler Viia7 (Applied Biosystems, Foster City, USA). PCR conditions were as follows: 95°C for 10 min, 45 cycles of 15 s at 95°C, and 1 min at 60°C. Reactions were performed in triplicate. Expression levels of the gene of interest were calculated in relation to the housekeeping gene (ΔCT = CT_target_ – CT_GAPDH_). Relative gene expression values are expressed as 2-(ΔΔCT), resulting from the difference between ΔCT_target_ - ΔCT_Calibrator_. DMSO-treated cells were used as calibrator.

### 2.5. Analysis of Senescence via Light Microscopy

Experiments were performed in 48-well plates replicated three times using the senescence *β*-galactosidase staining kit from Cell Signaling Technology, Inc. (Beverly, USA) according to the manufacturers' instructions. In brief, cells were washed, fixed in 1x fixative solution (10 min, RT), and incubated with *β*-galactosidase staining solution at 37°C at least overnight in a dry incubator. Detection of *β*-galactosidase-activity, visible as blue color, was performed with the light microscope Olympus CKX41 (Olympus GmbH, Hamburg, Germany) using a 10x objective.

### 2.6. Detection of Autophagy

Tumor cells were cultured on cover slips in 24-well plates (2.5 x 10^4^ cells/well). Upon treatment with the indicated substances/substance combinations, residual cells were stained with a mixture of acridine orange (4 mg/mL; Applichem, Darmstadt, Germany) and calcein AM (1.6 *μ*M; AAT Bioquest, CA, USA) for 10 min at RT according to the protocol described in [22]. Slides were analyzed on a laser scanning microscope (Zeiss) using 20x objectives.

### 2.7. Cell Cycle Analysis via Flow Cytometry

Treated cells and cell culture supernatants were harvested, collected in FACS tubes, and washed in 1x PBS (8 min, 200 x g). Then, supernatant was discarded and cells were resuspended in ice-cold ethanol (70 %), followed by incubation at -20°C for at least 12 hours. Afterwards, the cells were pelleted again by centrifugation (8 min, 200 x g), washed with 1x PBS and the cell pellet was resuspended in 1x PBS/0.1 % Tween 20 (Sigma-Aldrich Chemie GmbH, München, Germany)/RNase (1 mg/mL). Cells were incubated for at least one hour at -4°C. Following the addition of 50 *μ*g propidium iodide (PI, 0.1 mg/mL) (AppliChem GmbH, Darmstadt, Germany) cells were subjected to cytofluorometric analysis. Cell cycle analysis was performed on a BD FACS Calibur (BD) using the software Cell Quest Pro (BD). 10.000 events were measured for each sample. Data quantification was done by applying the BD ModFit LT Software for cell cycle analysis. Experiments were replicated three times.

### 2.8. Discrimination between Apoptosis and Necrosis via Flow Cytometry

Treated cells and cell culture supernatants were stained with YO-PRO-1 (0.2 *μ*M, 20 min, RT) and washed. PI (0.1 mg/mL) was added before performing the analysis on a BD FACS Calibur (BD). Cell staining and analysis was done as described [23].

### 2.9. Coculture with Immune Cells

Peripheral blood lymphocytes (PBLs) were obtained from healthy volunteers following pancoll (PAN-Biotech GmbH) density-gradient centrifugation. Tumor cells seeded in 24-well plates were treated with the test substances in the presence or absence of PBLs (ratio tumor cell to PBL 1:6). After 72 h of incubation, PBLs were removed by aspirating supernatant and remaining tumor cells were mixed with fluorescent beads (7x10^3^ beads/sample) (Fluoresbrite™Plain Beads, Polysciences Inc., Warrington, USA). PI (0.1 mg/mL) was added before performing the flow cytometric analysis on a FACSCalibur Cytometer (BD Pharmingen). Therefore, cells per 5,000 beads (= gate 1) were counted in the FSC/SSC dot plot. Cells cocultured with PBLs but without addition of test substances were used as control. Data analysis was performed using BD CellQuest™ Pro software.

### 2.10. Statistical Analysis

All values are expressed as mean ± SE. After proving the assumption of normality, differences between controls and experimental samples (=treated cells) were determined by using the unpaired Student's t-test (SigmaPlot 12.5). The criterion for significance was set to p < 0.05. Correlations were determined using Pearson correlation coefficient (r), with a range between -1 and +1. A value of 0 indicates that there is no association between the two variables. A value greater than 0 indicates a positive association (+1 = strong correlation).

## 3. Results

### 3.1. Phenotyping of Immune-Checkpoint-Molecules

**Figure 1:**
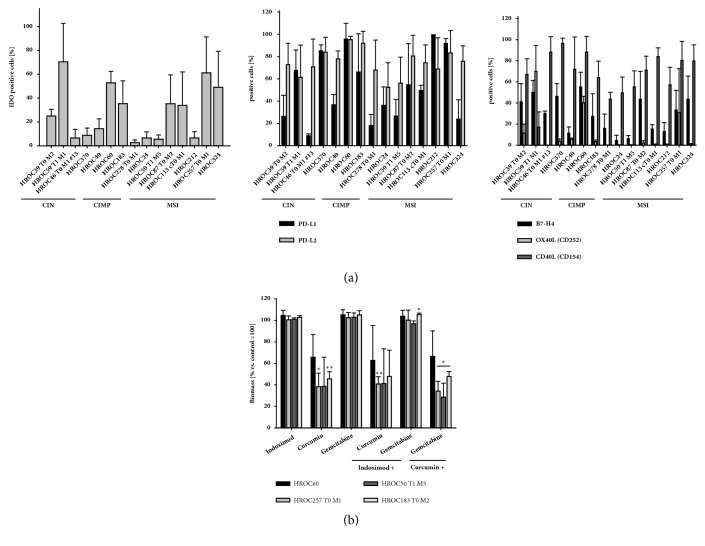
**Immunophenotyping and biomass quantification of tumor cells.** (a) Multicolor flow cytometry was conducted on a BD FACS Verse™ to examine the abundance of immune-related molecules as stated in material and methods. (b) Quantitative analyses of cell vitality using 0.2% crystal violet staining after 72 h incubation with test substances. Biomass reduction (%) after treatment was quantified by normalization to control values (untreated cells, set to be 100 %). N = 3 independent experiments, mean ± SD, *∗∗*p < 0.01; *∗*p < 0.05 versus control. Unpaired Student's* t*-test. CIN, chromosomal instable; CIMP, CpG island methylator phenotype; MSI, microsatellite instability/instable; PD-L1/PD-L2, programmed cell death 1 ligand 1/2; HROC, Hansestadt Rostock Colon.

The immune-phenotype of 17 low-passage CRC cell lines, covering the three main molecular subtypes, was characterized. All cell lines displayed high amounts of immunosuppressive molecules, such as PD-L1/2 (Figure 1(a)). Similarly, CTLA-4 and B7-H3 were detected in high abundance (always >90 %). IDO expression was heterogeneous among cell lines, ranging from < 5 % (HROC278 T0 M1) to > 60 % (HROC59 T1 M1). Contrary to that, costimulatory molecules were not detectable at all (i.e., CD27L, CD80, and CD86) with the exception of CD40L, which was heterogeneous. HROC40, HROC60, HROC183, and HROC113 T0 M1 cells were found to be MHC class I-positive and HROC257 T0 M1 showed positive staining for MHC class II. The remaining cell lines did not express MHC molecules. A comprehensive expression profile is given in Figure 1(a).

### 3.2. Functional Analysis of Substance-Mediated Growth Inhibition

On a basis of our phenotyping results, four cell lines with different IDO expression levels were chosen for further experiments: HROC257 T0 M1 (MSI, IDO^high^), HROC50 T1 M5 (MSI, IDO^low^), HROC60 (CIMP-L, IDO^high^), and HROC183 T0 M2 (CIMP-H, IDO^low^); they were treated with different substances (Indoximod, Curcumin, and Gemcitabine) reported to interfere with IDO [18, 24]. Drugs were applied for 72 h, either alone or in various combinations. Subsequently, biomass quantification was carried out (Figure 1(b)).

Indoximod alone did not impair cell viability significantly. Cytotoxic effects, if any, were detectable at high doses (> 20 *μ*M), but this did not reach statistical significance (*data not shown*). Of note, there was an inverse correlation between IDO abundance and response to high dose treatment with Indoximod (≥ 50 *μ*M). HROC50 T1 M5 responded best but showed lowest IDO expression in flow cytometry (35 % biomass reduction versus 5 % IDO1 expression,* data not shown*); conversely, HROC257 T0 M1 had high amounts of IDO1 (65 %) but did not respond to Indoximod-mediated growth inhibition (5 % biomass reduction versus control,* data not shown*). Curcumin had dose dependent cytotoxic activity (up to 80 % at 50 *μ*M). Gemcitabine was applied at subtherapeutic doses as determined in previous experiments [inhibitory concentration (IC) 10 [25, 26]] and chosen because of its promising antitumor activity when given in conjunction with other therapeutics.

Substance combinations enhanced antitumoral effects in almost all cases (Figure 1(b)). Best killing activity was obtained after treatment with Curcumin plus Gemcitabine with up to 70 % biomass reduction in MSI^+^ cell lines. CIMP-associated cell lines' response was weaker.

Sequential treatment (preincubation with Indoximod for 72 h) did not boost effects of the monotherapies. Thus, the direct cytotoxic potential of Indoximod on CRC cells was rather low. Even interferon-*γ* pretreatment, described to induce IDO expression and rendering cells more vulnerable to cytolysis [27], did not increase Indoximod-mediated growth inhibition (*data not shown*).

### 3.3. Gene Expression Analysis

**Figure 2:**
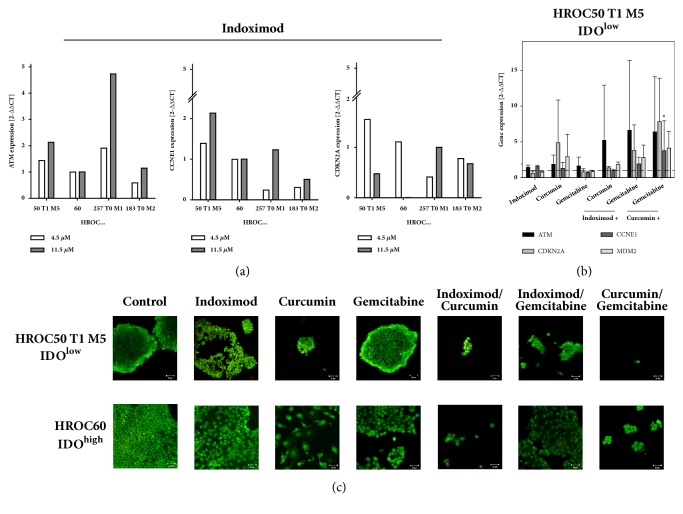
Quantitative gene expression analysis as determined by quantitative PCR (Taqman®). (a) Gene expression changes in HROC cell lines after Indoximod treatment (72 h, monotherapy). (b) Altered gene expression in HROC50 T1 M5 cells after combination with various test substances as stated in material and methods. Reactions were performed in triplicate wells and repeated three times. mRNA levels of target genes were normalized to the housekeeping gene* GAPDH*. The general expression level of each sample was considered by calculating 2^−ΔΔCT^ resulting from the difference between ΔCT_target_- ΔCT_Calibrator_. DMSO-treated cells were used as calibrator. N = 4 independent experiments, mean ± SD, *∗*p < 0.05 versus control. Unpaired Student's* t*-test. (c) Autophagy was detected by using confocal laser scanning microscopy. Cells were stained with acridine orange (excitation/emission: 500nm/526nm) to visualize acidic lysosomes within the cytoplasma, counterstaining was done with Calcein AM (excitation/emission: 494nm/517nm) as stated in material and methods. In most cases, autophagy was hardly detectable. Original magnification 200x. HROC, Hansestadt Rostock Colon.

Next, therapy-induced gene expression changes were analyzed. Indoximod monotherapy at a dose of 11.5 *μ*M led to a heterogeneous expression profile, with an increased expression of DNA-damage response gene* ATM* in MSI^+^ cell lines HROC257 T0 M1 and HROC50 T1 M5. Expression of* CDKN2A* and* CCNE1*, both being involved in cell cycle regulation, was largely unchanged (Figure 2(a)). Lower doses had no influence on gene expression at all (Figure 2(a)). Then, the gene expression profile of HROC50 T1 M5 cells after various treatments (i.e., Indoximod, Curcumin, Gemcitabine, Indoximod/Curcumin, Indoximod/Gemcitabine, and Curcumin/ Gemcitabine) was investigated to see if Indoximod-induced alterations are preserved or changed in the combination. Most significant changes were identified upon combined Curcumin/Gemcitabine treatment, resulting in a 5-fold induction of* ATM* and* CDKN2A* (p < 0.05 versus control).* CCNE1* and* MDM2* were also upregulated in this combination (Figure 2(b)).

### 3.4. Induction of Senescence by Test Substances

**Figure 3:**
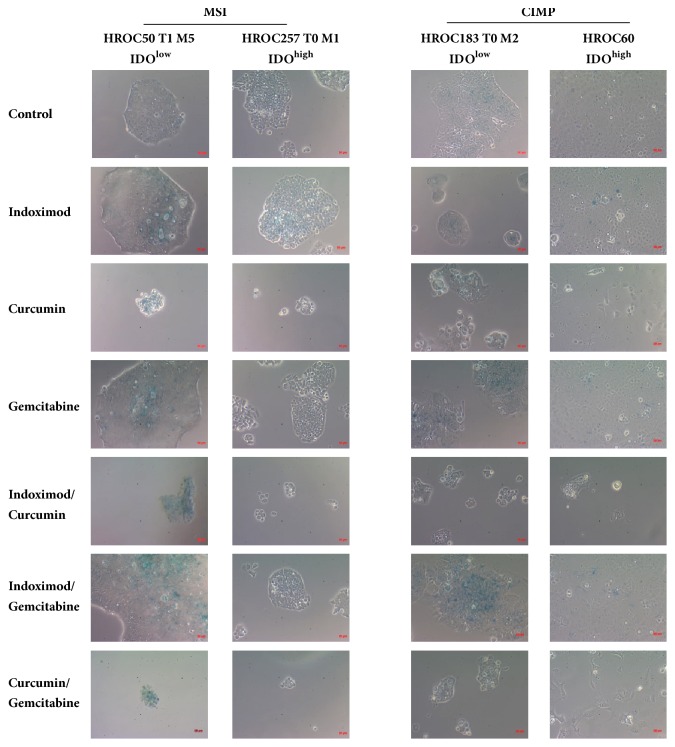
**Senescence detection by *β*-galactosidase staining.** Cells were incubated for 72 h with test substances and stained with X-Gal afterwards. Blue: ß-galactosidase-activity, indicative for senescence. Representative light microscopy images, original magnification 100 x. HROC, Hansestadt Rostock Colon; CIMP, CpG island methylator phenotype; MSI, microsatellite instability/instable.

We next aimed to unravel the underlying mechanisms of biomass reduction under treatment. Autophagy induction was studied by immunofluorescence based on staining of acidic vesicles with acridine orange. Frequency of autophagy was quite low (Figure 2(c)). By contrast, all substances and combinations caused senescence in HROC tumor cells with a tendency towards higher *β*-galactosidase activity after single treatment (Figure 3). Most pronounced effects were evident in HROC183 T0 M2 (IDO^low^) and HROC257 T0 M1 (IDO^high^) cells.

### 3.5. Altered Cell Cycle upon Treatment

**Figure 4:**
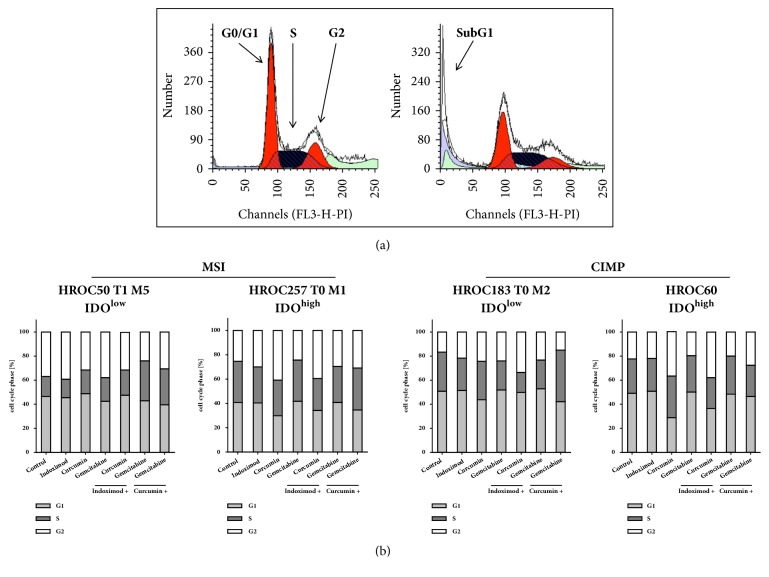
**Cell cycle analysis.** (a) Representative images showing apoptosis induction in Curcumin/Gemcitabine-treated HROC257 T0 M1 cells for 72 h as determined by flow cytometry and taking advantage of the BD ModFit LT Software. Cell number dependent on detected wavelength in nm (= DNA-content as an indicator for each cell cycle phase; Sub-G1: dead cells). Left: control; right: after Curcumin/Gemcitabine-treatment. (b) Quantitative cell cycle analysis of treated cell lines. Presented data result from % numbers of cells measured in each cell cycle phase (i.e., sum of G0/G1, S and M = 100%). Given are the mean values of N = 3 independent experiments. HROC, Hansestadt Rostock Colon; CIMP, CpG island methylator phenotype; MSI, microsatellite instability/instable.

Since senescence is associated with cell cycle arrest, flow cytometric Nicoletti staining was performed next. Almost all experimental conditions readily revealed an altered cell cycle accompanied by an increased amount of dead cells. This was most pronounced after Curcumin treatment, of note, both alone and in combination (Figure 4). Numbers of cells in G0/G1 phase decreased (HROC257 T0 M1 > HROC50 T1 M5 > HROC60 > HROC183 T0 M2; a representative example is given in Figure 4(a) showing Curcumin/Gemcitabine treated HROC257 T0 M1 (IDO^high^) cells; sub-G1 defines dead cells). The best antiproliferative effect was inducible in HROC183 T0 M2 (IDO^low^) cells after Indoximod/Curcumin treatment (Figure 4(b)). Percentages of cells in S-phase were 17 % ± 9 % versus 33 % ± 11 % (control).

### 3.6. Discrimination between Apoptosis and Necrosis

**Figure 5:**
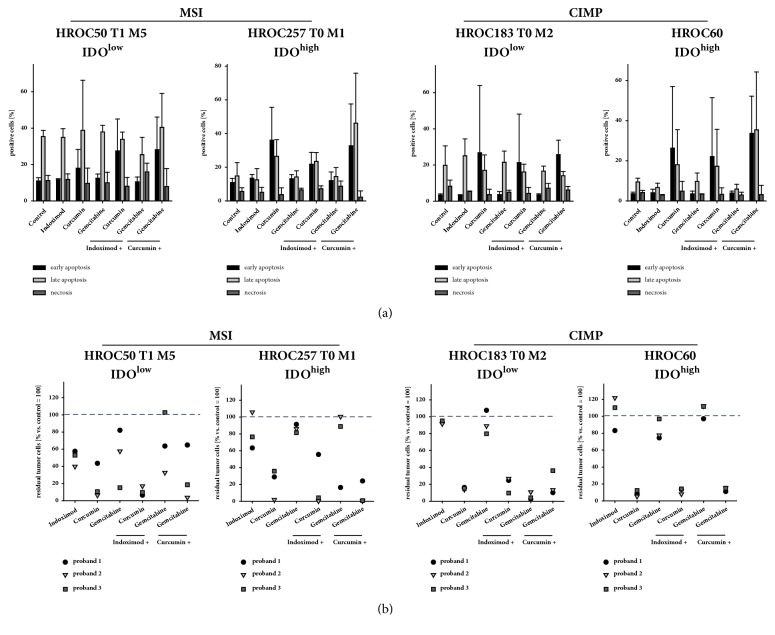
**(a) Apoptosis/necrosis discrimination and (b) coculture of tumor and immune cells.** (a) Quantitative analyses of cell death using flow cytometric Yo-Pro-1/PI staining after incubation with test substances for 72 h. For each sample, 10,000 events were measured. Dead cells were defined as early apoptotic (YO-PRO-1^+^), late apoptotic (YO-PRO-1^+^/PI^+^), or necrotic (PI^+^). Given are the % numbers of stained cells after treatment. N = 3 independent experiments, mean ± SD, *∗*p < 0.05 versus control. t-test. (b) Tumor and immune cells (PBL) from three healthy donors were coincubated in the presence or absence of test substances for 72 h. Tumor cell quantification was conducted by bead-based flow cytometry as stated in material and methods. Cells cocultured with PBLs but without addition of test substances were used as control. Percentage numbers of residual tumor cells for each donor are shown after 72 h and incubation with test substances. N = 3 PBL donor. HROC, Hansestadt Rostock Colon; CIMP, CpG island methylator phenotype; MSI, microsatellite instability/instable.

The above described findings identify cell death as main treatment effect. To test whether apoptosis, necrosis, or a mixture of both is responsible, Yo-Pro-1/PI staining was applied (Figure 5(a)). In all cases, apoptosis was the underlying mode of cell death (Figure 5(a)). Curcumin was most effective in inducing cellular apoptosis (at least 2-fold increase in all cell lines). In HROC257 T0 M1 (IDO^high^) cells, apoptosis was two to three times higher than in controls (early apoptosis: 36 ± 20 % versus 11 % ± 2 % control and late apoptosis: 26 % ± 10 % versus 15 % ± 8 % control). Adding Gemcitabine to Curcumin further enhanced cytotoxic effects (Figure 5(a)).

### 3.7. Coculture with Immune Cells Boosts Antitumoral Effects

IDO has differential effects on tumor and immune cells and thus can impair T-cell mediated tumor killing. In an* in vitro* coculture system, consisting of immune effector and tumor target cells, the potential of the different therapeutics to block IDO-induced negative immune effects was subsequently analyzed (Figure 5(b)).

All substances reduced tumor cell numbers in this test system. The best cytotoxic effect could be induced by Curcumin, resulting in a massive tumor cell reduction in all four cell lines (> 80 % versus control). Of note, combining Curcumin either with Indoximod or Gemcitabine even enhanced this toxic effect with nearly complete elimination of tumor cells (Figure 5(b)). Best tumor cell responder was the HROC60 (IDO^high^) cell line.

However, even with 72-hour incubation time, a specific antigenic activation is unlikely to occur and thus the observed effects are most likely due to a more unspecific stimulation of lymphocytes by the tested drugs.

## 4. Discussion

In this study, we describe (I) the expression profile of immune-modulating molecules on a panel of molecularly well-characterized patient-derived CRC cell lines and (II) the effects of IDO-inhibition directly on CRC cells as well as in coculture experiments with immune cells.

Phenotyping analyses revealed regularly high expression of immune-checkpoint-molecules PD-L1/2, B7-H3, B7-H4, and CD152 confirming the tumors' natural immunosuppressive character. IDO abundance was quite heterogeneous between cell lines and independent of their molecular phenotype. Studies on human CRC material similarly describe varying IDO expression* in vivo *[28]. Of note, abundance correlates inversely with the number of tumor-infiltrating CD3^+^ lymphocytes and clinical patients' outcome [29, 30].* In vitro*, IDO decreases tumor cell proliferation and mediates mediating drug resistance [31, 32]. While an association of cell growth with IDO expression was not obvious in our CRC lines (*data not shown*), we indeed found some overlap between IDO level and sensitivity towards clinically used drugs, such as 5-fluorouracil (5-FU; Pearsons* r* = 0.47, moderate positive relationship, Table 2). Hence, IDO plays a role in several pathways, making this molecule an interesting target for* in vitro* evaluation of a multimodal treatment strategy.

Specific IDO blockade with Indoximod identified an inverse correlation between biomass reduction and IDO1 abundance in our patient-derived cell lines. Of note, IDO^low^ expressing cells responded better towards this treatment, supporting recent findings that Indoximod likely targets additional pathways. The precise mechanism of action is not completely known. Indoximod was described to interfere with IDO translation and transcription as well as to inhibit the tryptophan-transporter in the plasma membrane [33]. Additionally, blocking IDO may sensitize tumor cells to certain chemotherapeutics and radiation by altering cell cycle (G1 → G2/M shift) [32]. However, in the present study, no chemosensitizing effects of Indoximod-“preconditioning” were detectable. Cytotoxic effects of Irinotecan, 5-FU, and Gemcitabine were also not boosted (*data not shown*).

The missing direct cytotoxic potential of Indoximod monotherapy* in vitro* encouraged us to use combinations with other IDO-blocking substances. Curcumin and Gemcitabine were selected based on the following criteria: (I) interference with IDO; (II) direct cytotoxic activity against tumor cells (via induction of immunogenic cell death) in (pre-)clinical studies, (III) safe application with minimal toxic side effects (at least in low-doses) in patients, and (IV) enhanced antitumoral effects when given in conjunction with other drugs. These experiments identified Curcumin as very promising candidate for IDO-blocking cancer therapy. Biomass of tumor cells significantly reduced and oncolytic effects even increased when Curcumin was combined with Gemcitabine, a quite surprising finding. In functional analyses, senescence and apoptosis could be identified as underlying molecular mechanisms. Effects were cell line and treatment specific, but again largely independent from IDO-expression status. Cytotoxicity of Curcumin is mainly based on apoptosis induction, without significant impairment of the cell cycle. This was somewhat unexpected, since short-time treatment with Curcumin has been reported to decrease cyclin D1 expression and to induce G1-/S-phase arrest [34, 35].

Finally, a coculture system mimicking tumor-immune cell interactions was applied to test the impact of IDO-inhibition for reconstitution of immune function.* In vivo*, IDO blockade by Indoximod breaks immune tolerance by increasing the level of inflammatory molecules, such as C-reactive protein and IL6 [10]. We could confirm such immune stimulating effects of Indoximod in 2/4 cell lines, but this was again rather independent of IDO-expression levels. Addition of Curcumin efficiently boosted the immune-mediated tumor cell elimination. This antitumoral effect is most likely attributable to unspecific immune stimulation by Curcumin and thus supports recent results of others [16, 36]. Gemcitabine, similar to Indoximod, also stimulated immune-mediated killing. Here again, NK cells are very likely the main effector cells [37]. However, effects were considerably lower than those of Curcumin. With the known toxic side effects of Gemcitabine, our findings argue in favor of Curcumin for further development of combinatorial treatment strategies.

Interestingly, there was a trend towards highest susceptibility of HROC60 cells, established from a 71-year-old male patient with a low-grade CIMP tumor in the* colon ascendens*. HROC60 cells show high abundance of immunosuppressive molecules, i.e., IDO, PD1/PD-L1. It is therefore of particular interest whether such Curcumin-based combinations provide a real opportunity for selected CRC patients in the future.

However, it is worth mentioning that coculture experiments were performed in an allogeneic setting using naïve and thus “unprimed” immune cells from healthy volunteers, without tumor-induced T-cell dysfunction. Prospective studies will have to test if comparable strong oncolytic effects can be achieved in an autologous setting with partially exhausted lymphocytes from cancer patients.

Although we were unsuccessful to directly link IDO-level with treatment response, we would like to bring forward the argument that this molecule plays a central role in tumor-induced immunosuppression. To this end, not only pharmacological agents, but also other means of IDO1 targeting may provide clinical benefits to patients. A recently performed phase I study even described disease stabilization in patients after application of an IDO1-peptide vaccine. In most patients with metastatic non-small cell lung carcinoma, detectable IDO-1-specific T cell responses were accompanied by significantly improved overall survival [38, 39]. Combining such vaccinations with Curcumin and/or low-dose chemotherapy [40] might increase their immune-stimulating capacity resulting in an efficient oncolytic regimen.

## 5. Conclusions

We report effects of IDO-inhibition on a panel of patient-derived colorectal cancer (CRC) cell lines. IDO blockade was induced by different substances, either directly (Indoximod) or indirectly (Curcumin, Gemcitabine) interacting with IDO. We have shown that Curcumin-based treatment is promising by inducing apoptosis in CRC cell lines, with even higher cytotoxic activity when Gemcitabine is added in low and thus nontoxic doses. Coculture of tumor and immune cells revealed a boosted antitumoral effect of Curcumin-based therapies by massive activation of unspecific immune responses, counteracting IDO-induced immune tolerance.

## Figures and Tables

**Table 1:** Clinical patients' information and molecular characterization of primary tumors used for cell line establishment.

				**T N M - classification**							**KRAS**			**TP53**				**APC**
**HROC**…	**age**	**gender**	**localization**	**T**	**N**	**M**	**G**	**molecular subtype**	**adjuvant chemotherapy**	**survival**	**Exon 2**	**Exon 3**	**BRAF**	**Exon 5**	**Exon 6**	**Exon 7**	**Exon 8**	
50 T1 M5	67	♀	Ascendens	T4	N0	M0	G2	spMSI	1st: Capecitabine; 2nd: 5-FU/LV	alive	wt	wt	V600E	wt	wt	N239K	wt	wt
257	84	♀	Ascendens	T4	N2	Mx	G3	spMSI	none	†, 84 J	wt	wt	V600E	n.a.	n.a.	n.a.	n.a.	n.a.
60	71	♂	Ascendens	T2	N0	M0	G2	CIMP-H	none	alive	wt	wt	wt	wt	wt	wt	R273H	Q1477*∗*
183	59	**♀**	Ascendens	T3	N2	M0	G3	CIMP-H	FOLFOX	alive	wt	wt	wt	R280W	R1450*∗*	wt	mut	wt

HROC, Hansestadt Rostock Colon; T, transfer, M - Mouse, Met, metastasis; 5-FU, 5-fluorouracil; LV, leucovorin; FOLFOX, 5-FU/LV/Oxaliplatin; *∗* May 2017, wt, wildtype; mut, mutated; n.a., not analyzed.

**Table 2:** Correlation between IDO abundance and drug response.

**cell line**	**IC**_**50**_			**IDO** **Expression (**%**)**
	** 5-FU**	**Cisplatin **	**Gemcitabine**	
	**[**μ**M]**	**[**μ**M]**	**[**μ**M]**	
**HROC24**	8.1	5.0	7.8	6.6
**HROC50 T1 M5**	2.3	n.a.	n.a.	5.6
**HROC87 T0 M2**	1.9	5.0	12.0	35.2
**HROC113 cT0 M1**	22.9	2.7	3.4	33.9
**HROC212**	n.a.	n.a.	n.a.	6.6
**HROC257 T0 M1**	19.3	9.0	10.8	61.2
**HROC324**	28.7	n.a.	n.a.	49.0
**HROC39 T0 M2**	5.0	7.3	3.4	25.0
**HROC59 T1 M1**	17.0	4.3	41.1	70.3
**HROC46 T0 M1 #13**	8.0	10.3	14.8	6.6
**HROC370**	4.0	n.a.	n.a.	8.8
**HROC40**	21.6	9.3	11.1	14.4
**HROC60**	7.7	6.3	6.7	52.8
**HROC183**	9.4	3.3	17.5	35.3
**HROC278 T0 M1**	n.a.	n.a.	n.a.	2.7

***Pearsons r***	**5-FU**	**Cisplatin**	**Gemcitabine**	
	**0.47**	**-0.28**	**0.49**	

HROC, Hansestadt Rostock Colon; 5-FU, 5-fluorouracil; IC, inhibitory concentration.

## Data Availability

The data used to support the findings of this study are available from the corresponding author upon request.

## Abbreviations

ATM: Ataxia Telangiectasia Mutated
CDKN2A: Cyclin-dependent kinase inhibitor 2A
CIN: Chromosomal instable
CIMP: CpG island methylator phenotype
CRC: Colorectal carcinoma
GAPDH: Glyceraldehyde-3-phosphate dehydrogenase
HRO: Hansestadt Rostock
IC: Inhibitory concentration
IDO: Indolamine 2,3-dioxygenase
LV: Leucovorin
MSI: Microsatellite instability/-instable
MHC I/II: Major histocompatibility complex I/II
MDM2: Murine double minute 2
NK: Natural killer
PBL: Peripheral blood lymphocytes
PD-1: Programmed cell death 1
PD-L1/PD-L2: Programmed cell death 1 ligand 1/2
PI: Propidium iodide
5-FU: 5-Fluorouracil.

## References

[1] Passardi A., Canale M., Valgiusti M., Ulivi P. (2017). Immune checkpoints as a target for colorectal cancer treatment. *International Journal of Molecular Sciences*.

[2] Sun X., Suo J., Yan J. (2016). Immunotherapy in human colorectal cancer: Challenges and prospective. *World Journal of Gastroenterology*.

[3] Le D. T., Durham J. N., Smith K. N. (2017). Mismatch repair deficiency predicts response of solid tumors to PD-1 blockade. *Science*.

[4] Turajlic S., Litchfield K., Xu H. (2017). Insertion-and-deletion-derived tumour-specific neoantigens and the immunogenic phenotype: a pan-cancer analysis. *The Lancet Oncology*.

[5] Johnson T. S., Mcgaha T., Munn D. H. (2017). Chemo-immunotherapy: Role of indoleamine 2,3-dioxygenase in defining immunogenic versus tolerogenic cell death in the tumor microenvironment. *Advances in Experimental Medicine and Biology*.

[6] Munn D. H., Mellor A. L. (2013). Indoleamine 2,3 dioxygenase and metabolic control of immune responses. *Trends in Immunology*.

[7] Zamanakou M., Germenis A. E., Karanikas V. (2007). Tumor immune escape mediated by indoleamine 2,3-dioxygenase. *Immunology Letters*.

[8] Liu M., Wang X., Wang L. (2018). Targeting the IDO1 pathway in cancer: from bench to bedside. *Journal of Hematology & Oncology*.

[9] Fox E., Oliver T., Rowe M. (2018). Indoximod: An immunometabolic adjuvant that empowers T cell activity in cancer. *Frontiers in Oncology*.

[10] Soliman H. H., Minton S. E., Han H. S. (2016). A Phase I study of indoximod in patients with advanced malignancies. *Oncotarget *.

[11] Soliman H., Khambati F., Han H. S. (2018). A phase-1/2 study of adenovirus-p53 transduced dendritic cell vaccine in combination with indoximod in metastatic solid tumors and invasive breast cancer. *Oncotarget *.

[12] Beatty G. L., O'Dwyer P. J., Clark J. (2017). First-in-human phase I study of the oral inhibitor of indoleamine 2,3-dioxygenase-1 epacadostat (INCB024360) in patients with advanced solid malignancies. *Clinical Cancer Research*.

[13] Xu B., Yu L., Zhao L.-Z. (2017). Curcumin up regulates T helper 1 cells in patients with colon cancer. *American Journal of Translational Research*.

[14] Milano F., Mari L., van de Luijtgaarden W., Parikh K., Calpe S., Krishnadath K. K. (2013). Nano-curcumin inhibits proliferation of esophageal adenocarcinoma cells and enhances the T cell mediated immune response. *Frontiers in Oncology*.

[15] Yin T.-F., Wang M., Qing Y., Lin Y.-M., Wu D. (2016). Research progress on chemopreventive effects of phytochemicals on colorectal cancer and their mechanisms. *World Journal of Gastroenterology*.

[16] Varalakshmi C., Ali A. M., Pardhasaradhi B. V. V., Srivastava R. M., Singh S., Khar A. (2008). Immunomodulatory effects of curcumin: In-vivo. *International Immunopharmacology*.

[17] Schafer C. C., Wang Y., Hough K. P. (2016). Indoleamine 2,3-dioxygenase regulates anti-tumor immunity in lung cancer by metabolic reprogramming of immune cells in the tumor microenvironment. *Oncotarget *.

[18] Jung I. D., Jeong Y.-I., Lee C.-M. (2010). COX-2 and PGE2 signaling is essential for the regulation of IDO expression by curcumin in murine bone marrow-derived dendritic cells. *International Immunopharmacology*.

[19] Gürlevik E., Fleischmann-Mundt B., Brooks J. (2016). Administration of gemcitabine after pancreatic tumor resection in mice induces an antitumor immune response mediated by natural killer cells. *Gastroenterology*.

[20] Lin X., Huang M., Xie F., Zhou H., Yang J., Huang Q. (2016). Gemcitabine inhibits immune escape of pancreatic cancer by down regulating the soluble ULBP2 protein. *Oncotarget*.

[21] Maletzki C., Huehns M., Knapp P. (2015). Functional characterization and drug response of freshly established patient-derived tumor models with cpg island methylator phenotype. *PLoS ONE*.

[22] Maletzki C., Rosche Y., Riess C. (2017). Deciphering molecular mechanisms of arginine deiminase-based therapy – Comparative response analysis in paired human primary and recurrent glioblastomas. *Chemico-Biological Interactions*.

[23] Maletzki C., Klier U., Marinkovic S., Klar E., Andrä J., Linnebacher M. (2014). Host defense peptides for treatment of colorectal carcinoma-a comparative in vitro and in vivo analysis. *Oncotarget *.

[24] Jeong Y.-I., Kim S. W., Jung I. D. (2009). Curcumin suppresses the induction of indoleamine 2,3-dioxygenase by blocking the Janus-activated kinase-protein kinase C*δ*-STAT1 signaling pathway in interferon-*γ*-stimulated murine dendritic cells. *The Journal of Biological Chemistry*.

[25] Maletzki C., Stier S., Gruenert U. (2012). Establishment, characterization and chemosensitivity of three mismatch repair deficient cell lines from sporadic and inherited colorectal carcinomas. *PLoS ONE*.

[26] Maletzki C., Gock M., Randow M. (2015). Establishment and characterization of cell lines from chromosomal instable colorectal cancer. *World Journal of Gastroenterology*.

[27] Ogawa K., Hara T., Shimizu M. (2012). (-)-Epigallocatechin gallate inhibits the expression of indoleamine 2,3-dioxygenase in human colorectal cancer cells. *Oncology Letters*.

[28] Brandacher G., Perathoner A., Ladurner R. (2006). Prognostic value of indoleamine 2,3-dioxygenase expression in colorectal cancer: Effect on tumor-infiltrating T cells. *Clinical Cancer Research*.

[29] Ferdinande L., Decaestecker C., Verset L. (2012). Clinicopathological significance of indoleamine 2,3-dioxygenase 1 expression in colorectal cancer. *British Journal of Cancer*.

[30] Ma W.-J., Wang X., Yan W.-T. (2018). Indoleamine-2,3-dioxygenase 1/cyclooxygenase 2 expression prediction for adverse prognosis in colorectal cancer. *World Journal of Gastroenterology*.

[31] Uyttenhove C., Pilotte L., Théate I. (2003). Evidence for a tumoral immune resistance mechanism based on tryptophan degradation by indoleamine 2,3-dioxygenase. *Nature Medicine*.

[32] Vareki S. M., Chen D., Di Cresce C. (2015). IDO downregulation induces sensitivity to pemetrexed, gemcitabine, FK866, and methoxyamine in human cancer cells. *PLoS ONE*.

[33] Moon Y. W., Hajjar J., Hwu P., Naing A. (2015). Targeting the indoleamine 2,3-dioxygenase pathway in cancer. *Journal for ImmunoTherapy of Cancer*.

[34] Singh A. K., Sidhu G. S., Deepa T., Maheshwari R. K. (1996). Curcumin inhibits the proliferation and cell cycle progression of human umbilical vein endothelial cell. *Cancer Letters*.

[35] Zhou Q.-M., Wang X.-F., Liu X.-J., Zhang H., Lu Y.-Y., Su S.-B. (2011). Curcumin enhanced antiproliferative effect of mitomycin C in human breast cancer MCF-7 cells in vitro and in vivo. *Acta Pharmacologica Sinica*.

[36] Bill M. A., Bakan C., Benson Jr. D. M., Fuchs J., Young G., Lesinski G. B. (2009). Curcumin induces proapoptotic effects against human melanoma cells and modulates the cellular response to immunotherapeutic cytokines. *Molecular Cancer Therapeutics*.

[37] Klier U., Maletzki C., Göttmann N., Kreikemeyer B., Linnebacher M. (2011). Avitalized bacteria mediate tumor growth control via activation of innate immunity. *Cellular Immunology*.

[38] Iversen T. Z., Engell-Noerregaard L., Ellebaek E. (2014). Long-lasting disease stabilization in the absence of toxicity in metastatic lung cancer patients vaccinated with an epitope derived from indoleamine 2,3 dioxygenase. *Clinical Cancer Research*.

[39] Vacchelli E., Aranda F., Eggermont A. (2014). Trial watch: IDO inhibitors in cancer therapy. *OncoImmunology*.

[40] Kanai M., Yoshimura K., Asada M. (2011). A phase I/II study of gemcitabine-based chemotherapy plus curcumin for patients with gemcitabine-resistant pancreatic cancer. *Cancer Chemotherapy and Pharmacology*.

